# Efficacy of Medical Treatment Options for Reducing the Risk of Bleeding in Patients With Sporadic and Familial Cerebral Cavernous Malformations: A Systematic Review and Network Meta-Analysis

**DOI:** 10.7759/cureus.116458

**Published:** 2026-09-17

**Authors:** Eren Gulsahin, Murat Durna, Okyanus Bora Gulec, Talha Eminhan Baser, Sam Elmuslimani, Secil Yerci, Irem Kaba, Riza Deha Dogruer, Berkay Kilic, Doga Damla Demir Yangi, Kivanc Yangi

**Affiliations:** 1 School of Medicine, Marmara University, Istanbul, TUR; 2 Department of General Practice, Kars Harakani Public Hospital, Kars, TUR; 3 School of Medicine, Akdeniz University, Antalya, TUR; 4 School of Medicine, Biruni University, Istanbul, TUR; 5 School of Medicine, Aydin Adnan Menderes University, Aydin, TUR; 6 School of Medicine, Ondokuz Mayıs University, Samsun, TUR; 7 School of Medicine, Pamukkale University, Denizli, TUR; 8 Gulhane School of Medicine, University of Health Sciences, Ankara, TUR; 9 School of Medicine, Istanbul University-Cerrahpaşa, Istanbul, TUR; 10 Department of Translational Neuroscience, Ivy Brain Tumor Center, Phoenix, USA; 11 Department of Translational Neuroscience, Barrow Neurological Institute, St. Joseph’s Hospital and Medical Center, Phoenix, USA; 12 Department of Neurosurgery, Barrow Neurological Institute, St. Joseph’s Hospital and Medical Center, Phoenix, USA

**Keywords:** antithrombotics, beta-blockers, bleeding risk, cerebral cavernous malformations, intracerebral hemorrhage​, network meta-analysis, statins

## Abstract

Cerebral cavernous malformations (CCMs) carry a significant hemorrhage risk. While surgery remains the primary treatment, deep-seated or eloquent lesions pose high operative risks, prompting interest in potential pharmacological approaches aimed at lesion stabilization. However, standardized medical management guidelines remain absent. This study evaluates the associations of statins, antithrombotics (ATs), and beta-blockers (BBs) with CCM hemorrhage risk.

Following the Preferred Reporting Items for Systematic Reviews and Meta-Analyses (PRISMA) guidelines (International Prospective Register of Systematic Reviews (PROSPERO): CRD420251069357), a systematic review of PubMed, Embase, Cochrane, and Scopus (inception to February 3, 2026) was conducted. Eligible studies compared bleeding outcomes in CCM patients receiving statins, ATs, or BBs versus no medical treatment. A multivariate meta-analysis utilized odds ratios (ORs) and surface under the cumulative ranking curve (SUCRA) scores to evaluate follow-up (re)bleeding (primary outcome) and hemorrhagic presentation at diagnosis (secondary outcome).

Seven studies (n = 3,092) were included. Statin therapy was associated with lower odds of follow-up (re)bleeding versus BBs (OR = 0.32, 95% confidence interval (CI): 0.11-0.95) and no treatment (OR = 0.32, 95% CI: 0.14-0.73). Triple combination therapy (statin + AT + BB) was also associated with lower odds of (re)bleeding compared with no treatment (OR = 0.24, 95% CI: 0.06-0.95) and ranked highest according to SUCRA. Statin + AT was associated with lower (re)bleeding odds versus BBs (OR = 0.25, 95% CI: 0.09-0.73). Conversely, BB monotherapy was associated with higher odds of (re)bleeding compared with AT monotherapy (OR = 2.41, 95% CI: 1.13-5.10). Untreated patients had higher odds of hemorrhagic presentation compared with statin + AT (OR = 3.10, 95% CI: 1.31-7.39) and AT monotherapy (OR = 1.86, 95% CI: 1.09-3.13). In the SUCRA ranking, statin + AT had the lowest ranked risk of hemorrhagic presentation at diagnosis.

In the absence of established pharmacological strategies, statin- and AT-based regimens were associated with lower CCM hemorrhage odds. However, the available evidence is predominantly observational and limited, with substantial potential for confounding by indication. Together with the null findings of the randomized AT CASH EPOC trial, these findings should be considered hypothesis-generating and are insufficient to guide clinical practice pending prospective randomized validation.

## Introduction and background

Cerebral cavernous malformations (CCMs) are low-flow, low-pressure clusters of dilated, endothelium-lined vascular caverns [1,2]. These lesions are the second most common incidental vascular finding on brain magnetic resonance imaging (MRI) after aneurysms, with a prevalence of approximately 0.1%-0.5% [2,3].

CCMs can occur in either sporadic or familial forms. Sporadic CCMs typically present as a single lesion, whereas the familial forms commonly involve multiple lesions and account for approximately 40%-60% of cases [4]. Familial CCMs are generally caused by mutations in the CCM1 (KRIT1), CCM2 (MGC4607), and CCM3 (PDCD10) genes [4,5]. CCMs can occur throughout the brain, with 70%-80% located supratentorially; these tend to present with new-onset seizures, whereas infratentorial lesions more often cause progressive neurological deficits [4]. They are prone to microhemorrhages, thrombosis, and rupture, leading to seizures, headaches, and hemorrhagic stroke [6,7]. The annual hemorrhage risk is estimated at ~0.7%-1.1% per lesion in the absence of prior bleeding, rising to ~4.5% after a first hemorrhage, and is generally higher for brainstem CCMs [2,4,8].

Treatment decisions in CCMs are guided by lesion location, symptom severity, and lesion burden (e.g., solitary vs multiple). For symptomatic, surgically accessible lesions, microsurgical resection is generally the first-line treatment. In contrast, small asymptomatic lesions are often managed with observation and interval imaging. In selected patients, particularly when lesions are deep-seated or high-risk for open surgery, radiosurgery may be considered as an alternative option [7,9].

Given the potential risks of current treatment modalities, there has been growing interest in recent years in whether medical therapies might stabilize CCMs and reduce the risk of hemorrhagic events [10-13]. Advances in understanding CCM pathophysiology have prompted pharmacological investigations, including antithrombotics (ATs), beta-blockers (BBs), and statins, with observational studies reporting associations between AT exposure and lower hemorrhage risk [9,10,14]. BBs, particularly propranolol, have also been investigated in CCM management, with some studies reporting potentially favorable associations [15]. Statins are also of considerable interest as potential stabilizing agents [11]. However, the available evidence remains limited and heterogeneous, and the predominantly observational nature of these studies leaves uncertainty regarding treatment effects and susceptibility to confounding. This study synthesizes the available clinical evidence regarding exposure to statins, BBs, and ATs in patients with sporadic and familial CCMs using a network meta-analysis framework. This approach enables the integration of direct and indirect evidence across multiple treatment strategies, allowing comparative estimation even when head-to-head comparisons are limited or unavailable. The aim was to evaluate associations between these pharmacological strategies and hemorrhagic outcomes, explore comparative treatment rankings, and identify priorities for future bleeding-prevention trials.

This work was previously accepted as an abstract at the North American Skull Base Society (NASBS) Annual Meeting (March 5-8, 2026) and was presented as a poster by the senior author (K.Y.). The abstract is available at https://doi.org/10.1055/s-0046-1819393

## Review

Methods

Study Design

This study was conducted in accordance with the Preferred Reporting Items for Systematic Reviews and Meta-Analyses (PRISMA) [16], and the study protocol was registered with the International Prospective Register of Systematic Reviews (PROSPERO) under the registration number CRD420251069357.

Eligibility Criteria

Inclusion criteria: Studies were included if they met the predefined patient criteria, including patients who presented either incidentally, with first-time symptomatic hemorrhage, or with a history of recurrent bleeding, all accompanied by a diagnosis of sporadic or familial CCMs confirmed by imaging (susceptibility-weighted MRI (SWI) or T2-weighted sequences), which served as the diagnostic gold standard [17]. Eligible studies enrolled patients who received systemic administration of pharmacological agents, including ATs (aspirin and/or clopidogrel), statins (atorvastatin, simvastatin, rosuvastatin, pravastatin, or fluvastatin), and/or BBs (mostly propranolol). The outcomes of interest were the provable clinical endpoints, including hemorrhage at presentation and symptomatic hemorrhage during follow-up. Eligible study designs included randomized controlled trials (RCTs), prospective or retrospective cohort studies with an appropriate comparator group (including those suitable for indirect comparisons), and case-control studies with comparative data amenable to indirect comparison. Studies were required to provide sufficient information to permit direct or indirect treatment comparisons within a network meta-analysis framework.

Exclusion criteria: Studies were excluded if they focused exclusively on spinal cavernous malformations (CMs) or reported mixed spinal and cerebral cases in aggregate without providing separate cerebral-specific data, precluding independent analysis. We also excluded studies investigating vascular malformations other than CCMs. Additionally, studies involving surgical procedures, stereotactic radiosurgery, radiotherapy-based interventions, or local/non-systemic drug administration without post-intervention censoring were deemed ineligible. We excluded non-English publications, case reports, narrative reviews, expert opinions, and case series without comparators. Studies lacking direct or indirect comparisons, failing to report predefined outcomes, or using unclear or non-validated outcome definitions were also excluded. Additionally, studies limited to surgical or procedural outcomes without assessment of pharmacological interventions, as well as animal or in vitro studies, were excluded.

Search Strategy

A systematic literature search was conducted independently by two reviewers (E.G. and K.Y.) across PubMed, Embase, Cochrane, and Scopus databases from inception to February 3, 2026. The search strategy included the following terms: bleeding OR hemorrhage AND cerebral cavernous malformation AND medical treatment OR antithrombotic OR statin OR acetylsalicylic acid OR beta-blocker. These terms were combined using Boolean operators ‘’AND’’ and ‘’OR’’ to ensure maximum comprehensiveness. The full search strategy is provided in Appendix A. The reviewers screened the extracted studies for relevance to the topic of interest and provided an overall assessment of the adequacy and completeness of the available literature. Additionally, citation search and manual search methods were conducted. All retrieved records were then uploaded to Rayyan [18] for deduplication, screening, and study selection.

Study Selection and Data Extraction

The study selection process was conducted by independent reviewers in two stages: title and abstract screening (I.K., K.Y.) and full-text screening (O.B.G., K.Y.) using Rayyan [18]. Each stage was conducted independently and subsequently cross-checked by two reviewers. In the case of multiple publications of the same study, only the latest version was included. Additionally, two independent reviewers (D.D.D.Y. and K.Y.) checked the reference lists of all the included articles. Any disagreements were resolved through discussion or, if necessary, by consulting a third reviewer until a consensus was reached at each stage. Subsequently, data extraction was performed using a standardized form designed in Microsoft Excel (Microsoft Corporation, Redmond, WA, USA) by three independent reviewers (S.E., D.D.D.Y., and B.K.). A fourth reviewer (K.Y.) reviewed the extracted data for consistency and adjudicated any outstanding discrepancies.

Treatment exposure was documented based on the exposure criteria reported in each included study. When available, information regarding the pharmacological class as well as the timing and duration of medication use was extracted. Because exposure ascertainment differed across the included studies, information on the timing and duration of treatment was not uniformly available.

Outcome Measures

The primary outcome of the study was the occurrence of prospective (re)bleeding during follow-up in patients with CCMs. No predefined minimum or maximum follow-up period was specified. Follow-up duration was recorded as reported in the original studies and summarized in person-years for descriptive purposes; when required, person-years were approximated or calculated as described in Table 1. The secondary outcome was the association between prior medication use and hemorrhagic presentation at the time of CCM diagnosis. Hemorrhagic outcomes were extracted according to the definitions provided by the original studies, provided that they were consistent with the eligibility criteria of the present review. CCM-related hemorrhages were defined as new symptomatic events, such as focal neurologic deficits (FNDs), seizures, and acute or subacute onset of headache, as well as asymptomatic events confirmed by radiological, pathological, surgical, or autopsy evidence.

**Table 1:** Baseline methodological and clinical characteristics of included studies ^a^Approximated from lesion-years ^b^Non-contiguous recruitment; two epochs, no enrollment in 2004-2005 ^c^Calculated from median (IQR) age using the formula from Wan et al. [25] ^d^Reported as patient number with supratentorial/infratentorial lesion ^e^Estimated as the area under the Kaplan-Meier survival curve (restricted mean survival time) to 24 months, using the published numbers-at-risk and converting person-days to years by dividing by 365.25 using the formula from Royston and Parmar [26] ^f^Values represented as n, supratentorial and infratentorial, CCM lesion per patient with data for the variable, respectively CC: case-control; FUD: follow-up duration; N/A: non-applicable; N/S: not specified; PC: prospective cohort; RC: retrospective cohort; RCS: retrospective cross-sectional; RCT: randomized controlled trial; SD: standard deviation

Author, year	Study design	Country	Recruitment window	Patients/lesions (n/n)	Sex (male/female) (n (%)/n (%))	Age, mean (SD)	Lesion (supratentorial/infratentorial) (n/n)	Patients (single/multiple lesions) (n/n)	Patients (sporadic/familial) (n/n)	FUD (person-years)
Marques et al., 2023 [19]	RC	Switzerland	1980-2021	688/933	367 (53.3%)/321 (46.7%)	51.3 (18.7)	696/237	592/96	N/S	2,569^a^
Gomez-Paz et al., 2020 [20]	RCS	USA	1990-2018	446/635	216 (48.4%)/230 (51.6%)	50 (17)	441/194	382/64	N/S	N/A
Zuurbier et al., 2022 [11]	PC	Scotland	1999-2003, 2006-2010^b^	300/N/S	141 (47%)/159 (53%)	44.5 (17)^c^	N/S	203/97	N/S	3,634
Chen et al., 2023 [21]	RC	Germany	2003-2021	1,116/1,116	433 (38.8%)/683 (61.2%)	42 (15.9)	722/394^e^	1,116/0	1,116/0	2,642
Santos et al., 2022 [22]	RC	Germany	2003-2020	205/N/S	88 (42.9%)/117 (57.1%)	34.62 (18.58)	N/S	N/S	0/205	534
Awad et al., 2025 [23]	RCT	USA	2018-2022	80/N/S	29 (36.3%)/51 (63.7%)	42 (12.8)^c^	32/48^d^	51/29	51/29	132.7^e^
Scerrati et al., 2023 [24]	CC	Italy	N/S	257/N/S	120 (46.7%)/137 (53.3%)	43.4 (12.5)^c^	181/72^f^	161/96	N/S/15	944.7^c^

Risk-of-Bias Assessment

The included studies were independently evaluated for internal validity by two reviewers (R.D.D. and K.Y.). Five observational studies were assessed using the Newcastle-Ottawa Scale (NOS) [27], which examines three domains (selection, comparability, and outcome), and were subsequently rated as good, fair, or poor according to their total scores. One cross-sectional study was assessed using the National Institutes of Health (NIH) Quality Assessment Tool [28]. Unlike the NOS-assessed studies, no numerical scoring was applied; instead, the study was qualitatively rated as good, fair, or poor based on the responses to the questions (yes, no, cannot determine, not reported, or not applicable). Furthermore, the Cochrane Risk of Bias 2 (RoB2) tool [29] was used for the systematic evaluation of one study, where five domains were assessed, with green (+) indicating low risk of bias, yellow (!) indicating some concerns, and red (-) indicating high risk of bias. Any discrepancies between the reviewers were resolved through discussion and consensus after cross-checking the data.

Assessment of Class of Evidence and Strength of Recommendations

The class of evidence and strength of recommendations of the included studies were graded according to the American Association of Neurological Surgeons/Congress of Neurological Surgeons (AANS/CNS) criteria for therapeutic evidence as detailed in Appendix B [30]. Two reviewers (E.G. and K.Y.) classified each study based on its design and the presence of a comparison group. Subsequently, the strength of recommendations (Level I, II, or III) was assigned based on the highest level of supporting evidence [30].

Statistical Analysis

Effect estimates were extracted from the included studies for the network meta-analysis. When both adjusted and unadjusted effect estimates were reported, the adjusted estimate was used; when an adjusted estimate was unavailable, the unadjusted estimate was included. Adjustment variables differed across studies and were therefore not standardized across the network. Treatment exposure was classified into eight pharmacological strategies according to the reported medication regimens. No imputation was performed for missing outcome data; analyses were based on the available outcome data reported in the included studies.

Both randomized and non-randomized studies were included within the same network because randomized comparative evidence was limited, and both study designs provided eligible data for the pre-specified hemorrhagic outcomes. Differences in study design were explicitly considered in the qualitative assessment of transitivity and in the interpretation of the network estimates.

For the network meta-analysis, odds ratios (ORs) were analyzed on the natural logarithmic scale. Study-level log ORs and their corresponding variance-covariance information were synthesized within a frequentist network meta-analysis framework, and pooled estimates were subsequently exponentiated and reported as conventional ORs with 95% confidence intervals (CIs). Patients receiving no pharmacological treatment of interest served as the principal reference group, while the network structure enabled both direct and indirect comparisons among the included treatment strategies. Statistical inconsistency within the treatment network was evaluated using a Wald-type chi-squared test [31].

The transitivity assumption was assessed qualitatively by comparing available clinical and methodological characteristics across studies contributing to the treatment comparisons. These included age, sex, hemorrhagic presentation or prior intracerebral hemorrhage, lesion location (particularly brainstem, deep, or infratentorial location), sporadic versus familial CCM status, lesion multiplicity, study design, treatment-exposure definition and timing, and follow-up structure and duration. These characteristics were considered as potential effect modifiers when evaluating the comparability of patient populations across treatment comparisons and the validity of indirect comparisons. Because these characteristics were not uniformly reported and differed across the included studies, transitivity could not be established definitively; indirect comparisons were therefore interpreted cautiously. Additional subgroup or sensitivity analyses were not performed because of the limited number of studies and sparse treatment comparisons within the network.

Interventions were ranked using the surface under the cumulative ranking curve (SUCRA). SUCRA values range from 0 (0%) to 1 (100%), with higher values indicating a greater probability of occupying a higher position in the treatment ranking. These rankings were interpreted as probabilistic treatment hierarchies rather than evidence of definitive clinical superiority. All statistical analyses were performed using the mvmeta and network packages in STATA version 16.0 (StataCorp LP, College Station, TX, USA).

Results

Study Selection

The initial search identified 1,426 publications. After removal of duplicates and ineligible studies, 28 studies remained for further evaluation. After full-text screening, 21 studies were excluded for failing to meet the eligibility criteria. Consequently, seven studies satisfied all inclusion criteria and were included in the final analysis (Figure 1).

**Figure 1:**
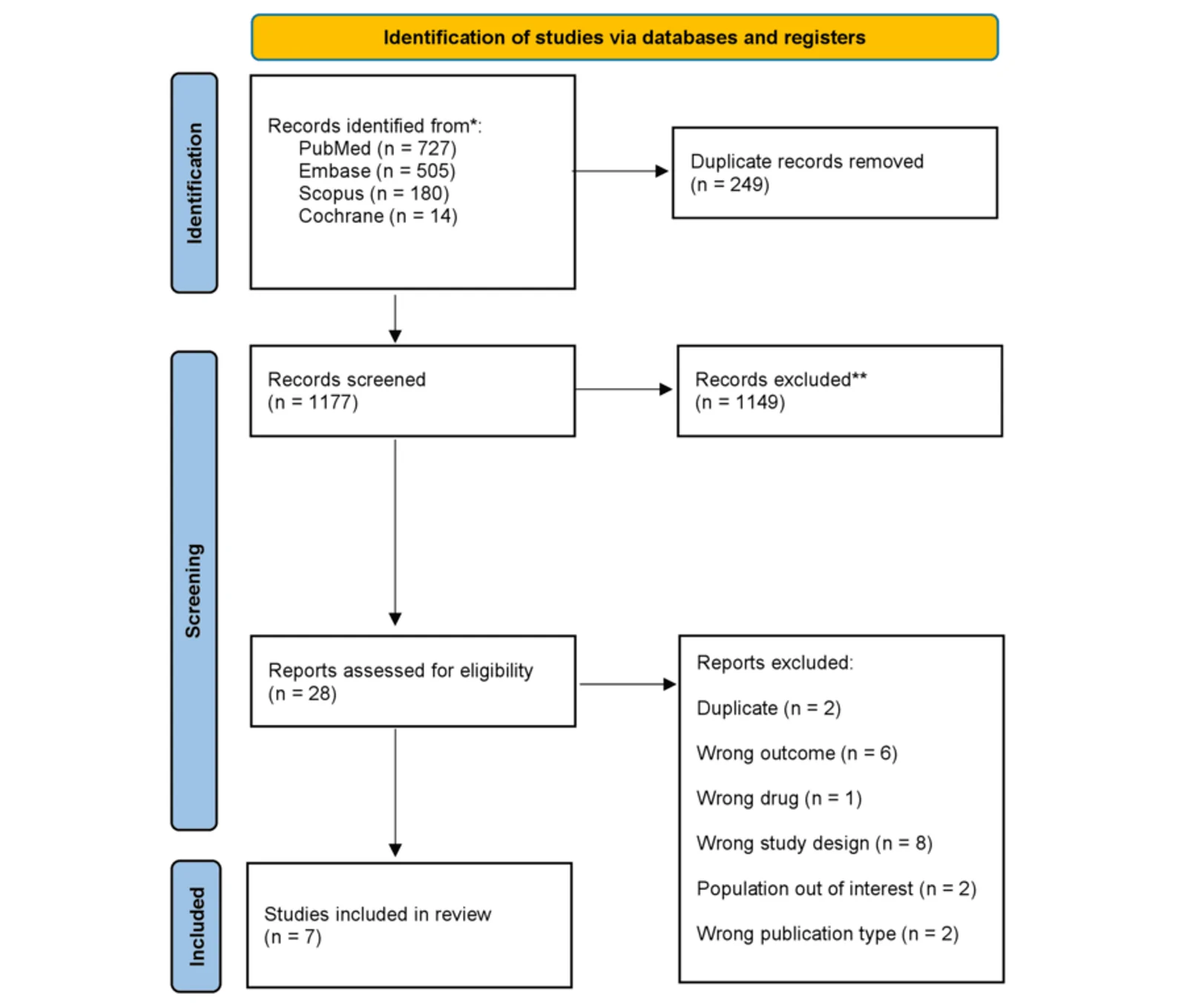
PRISMA flow diagram of the study selection process PRISMA: Preferred Reporting Items for Systematic Reviews and Meta-Analyses

Study Characteristics

Seven studies published between 2020 and 2025, including a total of 3,092 patients (mean age 45.1 ± 17.2 years, 54.90% women, 45.10% men) with sporadic or familial CCM, contributed data on eight treatment strategies: no medical therapy, statin monotherapy, AT monotherapy, BB monotherapy, and four different combinations of these agents (statin + AT, statin + BB, AT + BB, and statin + AT + BB) were analyzed. A total of 3,017 CCMs were reported. Two thousand seventy-two (68.7%) lesions were located in the supratentorial region, while 945 (31.3%) were infratentorial. In four studies, patient-level presentation was available, with 1,167 patients classified as sporadic and 249 as familial. Out of a total of 3,092 patients, 2,505 had single lesions, whereas 382 had multiple lesions. In the studies by Zuurbier et al. [11], Santos et al. [22], Awad et al. [23], and Scerrati et al. [24], the total number of CCM lesions was not explicitly reported. Although single and multiple lesion categories were provided with corresponding numerical values in the studies by Zuurbier et al. [11], Awad et al. [23], and Scerrati et al. [24], these figures were presented (or assumed) as the number of patients with single or multiple lesions, and their sums equaled the total study population rather than the absolute lesion count (Table 1).

Regarding treatment, 283 patients reported statin use, 251 reported AT therapy, and 198 reported use of BBs. Among patients receiving these treatments, 152 presented with hemorrhage at baseline. Subsequent hemorrhage occurred in 70 patients with available follow-up data (Table 2).

**Table 2:** Treatment group allocation and clinical outcome measures, with hemorrhagic presentation at diagnosis and subsequent hemorrhage during follow-up ^a^Values represent the number of patients receiving only this medication among S, AT, and BB ^b^Reported per lesion ^c^All patients experienced symptomatic hemorrhage within 1 year prior to the study ^d^Values given only for receiving antiplatelet medication Outcome data are reported as available in the original studies and may include event counts, ORs, HRs, 95% CIs, p-values, lesion-years, and incidence estimates. AT: antithrombotic; BB: beta-blocker; CI: confidence interval; combined: statin + antithrombotics; HR: hazard ratio; N/A: non-applicable; N/S: not specified; OR: odds ratio; P: placebo; S: statin

Study label	Drug group (n)	Comparison group (n)	Outcome	
			Hemorrhage at diagnosis	Hemorrhage during follow-up
Marques et al., 2023 [19]	S (45)^a^	No S or AT (545)	11^b^; OR, 0.63 (0.23-1.69); p = 0.355	0^b^; 100.6 lesion-years
	AT (46)^a^	No S or AT (545)	6^b^; OR, 0.26 (0.08-0.86); p = 0.028	2^b^; 137.1 lesion-years; 5-year incidence, 7.3%
	Combined (52)	No S or AT (545)	6^b^; OR, 0.19 (0.05-0.66); p = 0.009	0^b^; 100.7 lesion-years
Gomez-Paz et al., 2020 [20]	S (54)^a^	No S or AT (328)	14; OR, 0.71 (0.35-1.45); p = 0.347	N/A
	AT (20)^a^	No S or AT (328)	5; OR, 0.78 (0.23-2.60); p = 0.684	
	Combined (44)	No S or AT (328)	7; OR, 0.25 (0.10-0.61); p = 0.030	
Zuurbier et al., 2022 [11]	S (73)	No S (227)	13; OR, 1.04 (0.52-2.09); p = 0.902	4; HR, 0.75 (0.23-2.43); p = 0.632
	AT (61)	No AT (239)	N/S; N/A	1; HR, 0.12 (0.02-0.88); p = 0.037
	BB (63)	No BB (237)	4; OR, 0.27 (0.09-0.77); p = 0.010	1; HR, 0.09 (0.01-0.65); p = 0.017
Chen et al., 2022 [21]	S (54)	No S (1,062); no S or AT or BB (N/A)	20; OR, 1.273 (0.584-2.774); p = 0.543	18; HR, 0.457 (0.131-1.593); p = 0.219
	AT (62)	No AT (1,054); no S or AT or BB (N/A)	17; OR, 0.517 (0.255-1.047); p = 0.067	13; HR, 0.729 (0.214-2.486); p = 0.614
	BB (89)	No BB (1,027); no S or AT or BB (N/A)	42; OR, 0.683 (0.361-1.292); p = 0.241	21; HR, 1.140 (0.551-2.357); p = 0.724
Santoset al., 2022 [22]	S (16)	No S (143)	N/S; OR, 0.87 (0.29-2.60); p = 0.798	1; HR, 0.22 (0.03-1.68); p = 0.143
	AT (13)	No AT (109)	N/S; OR, 0.37 (0.11-1.30); p = 0.121	0; N/A
	BB (10)	No BB (148)	N/S; OR, 0.92 (0.36-2.34); p = 0.858	3; HR, 0.80 (0.23-2.80); p = 0.729
Awad et al., 2025 [23]	S (41)	P (39)	N/S^c^; N/A	6; HR, 0.77 (0.26-2.29); p = 0.729
Scerrati et al., 2023 [24]	AT (49)	No AT (208)	4; OR, 0.39 (0.07-2.12); p = 0.300^d^	N/A
	BB (36)	No BB (221)	3; OR, 1.24 (0.60-2.57); p = 0.600	

Quality Assessment

Risk of bias and overall network structure: Risk-of-bias assessment showed overall moderate-to-high methodological quality. Three cohort studies achieved 8-9 stars. Two studies received 5-7 stars, as shown in Appendix C. One additional study was evaluated with the NIH tool and judged to have clearly defined populations, valid exposure and outcome assessment, and appropriate control of confounders, although limitations remained regarding sample size justification and follow-up duration, as detailed in Appendix D. Additionally, the risk of bias in Awad et al. [23] was systematically evaluated using the Cochrane RoB2 tool. The detailed assessment is provided in Appendix E, where green (+) denotes low risk of bias, yellow (!) indicates some concerns, and red (-) reflects high risk of bias.

The geometry of the treatment network was well connected for both outcomes, with "No treatment" as the largest node and sharing the thickest edges with statin, AT, and combination regimens (Figures 2, 3). The global inconsistency tests for the multivariate network meta-analysis were non-significant for (re)bleeding during follow-up (χ²(7) = 4.30, p = 0.74) and for hemorrhagic presentation at diagnosis (χ²(6) = 5.89, p = 0.44), with no statistically detectable global inconsistency identified for either network.

**Figure 2:**
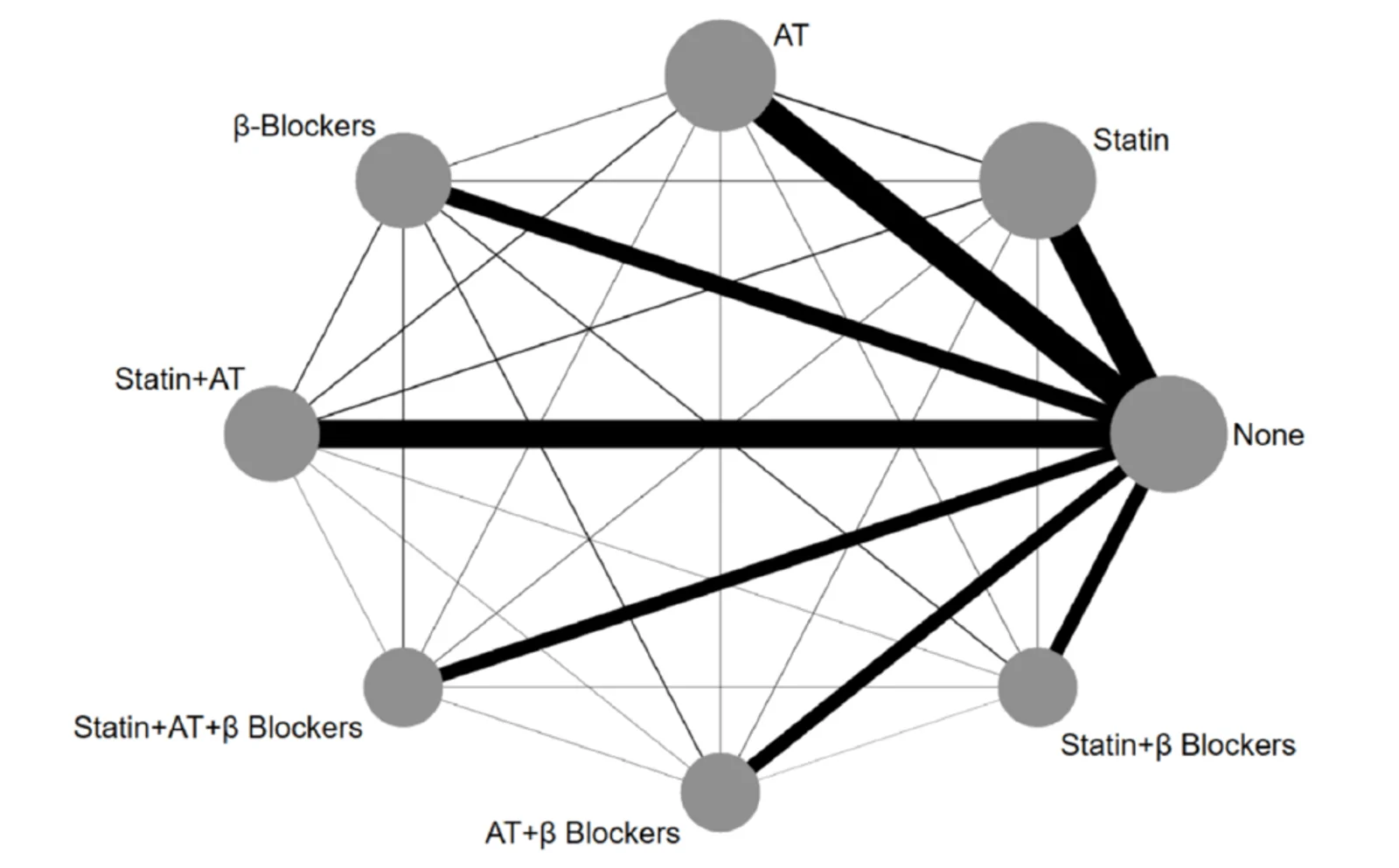
Network geometry of available clinical evidence for prospective (re)bleeding during follow-up The network plot maps the direct comparisons available across different single and combined pharmacological regimens. Each node represents a distinct pharmacological strategy, with the node size proportional to the total number of patients assigned to that treatment across the included studies. The solid lines (edges) connecting the nodes represent direct comparisons reported across the included studies, while the thickness of the edges reflects the relative volume of evidence contributing to each direct comparison. “None” (no treatment) represents the largest node and serves as a central comparator within the network. The largest volume of comparative evidence is observed between the no-treatment group and statin, AT, and statin + AT strategies. The figure illustrates the structure and relative amount of available comparative evidence rather than the magnitude or statistical significance of treatment associations. AT: antithrombotic The figure was created by the authors using STATA version 16 (StataCorp LP, College Station, TX, USA).

**Figure 3:**
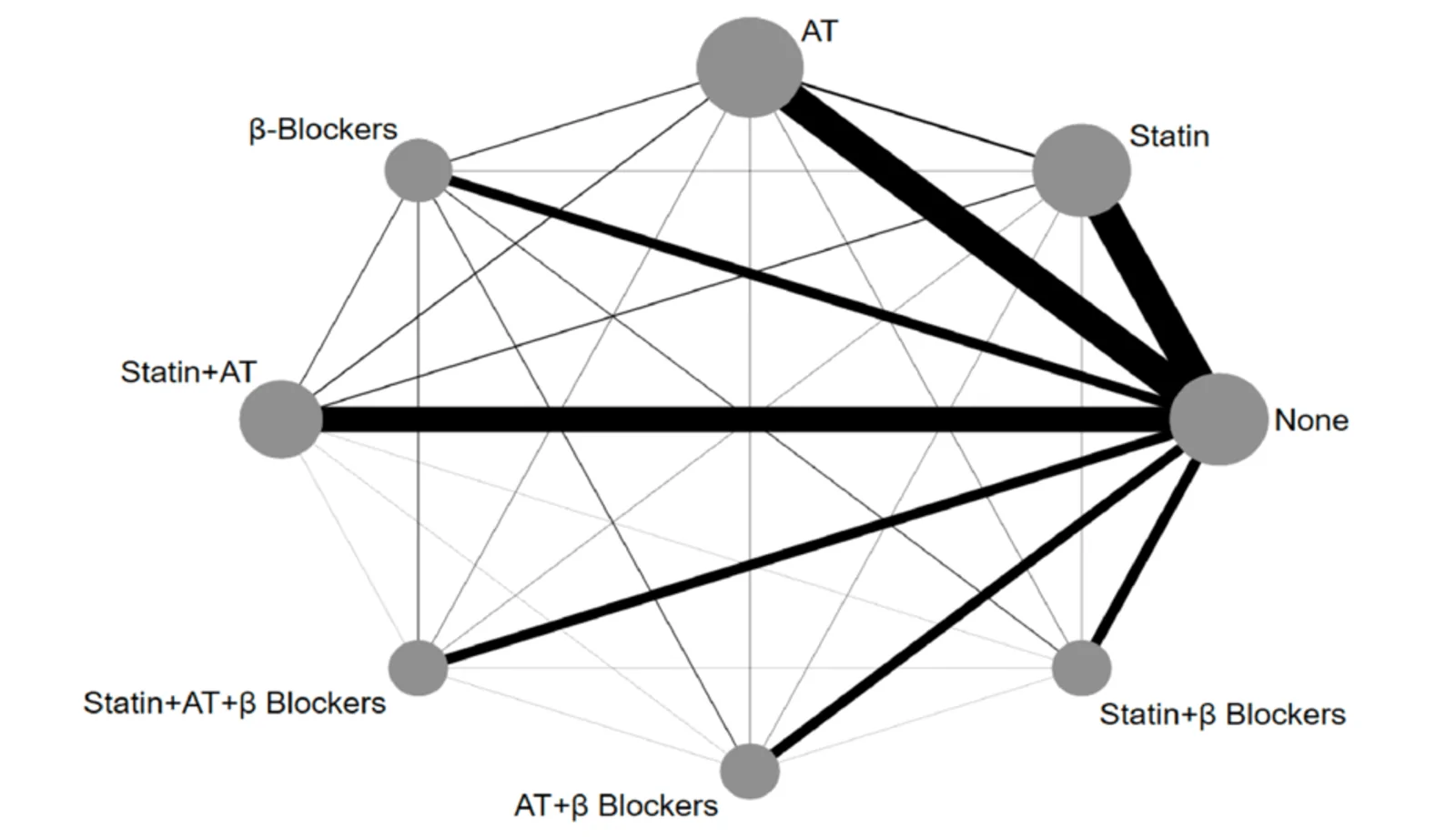
Network geometry of available clinical evidence for hemorrhage at presentation The network plot maps the direct comparisons available across different single and combined pharmacological regimens. Each node (circle) represents a distinct pharmacological strategy, with the node's size being proportional to the total number of patients assigned to that specific treatment arm across the included studies. The solid lines (edges) connecting the nodes represent direct comparisons reported across the included studies, while the thickness of the edges reflects the relative volume of evidence contributing to each direct comparison. “None” (no treatment) represents the largest node and serves as a central comparator within the network. The largest volume of comparative evidence is observed between the no-treatment group and single therapies such as AT and statin, as well as the statin + AT combination. Comparisons among active pharmacological strategies are supported by relatively less direct evidence. The figure illustrates the structure and relative amount of available comparative evidence rather than the magnitude or statistical significance of treatment associations. AT: antithrombotic The figure was created by the authors using STATA version 16 (StataCorp LP, College Station, TX, USA).

Grading Evidence and Levels of Recommendations

According to the AANS/CNS criteria provided in Appendix B, one study met the Class I criteria since it was an RCT [23]. Five prospective/retrospective comparative studies and one case-control study met the criteria for Class II evidence with a Level II recommendation [11,19-22,24].

Meta-Analysis

Primary outcome: Pooled data from seven included studies indicated that statin monotherapy was associated with significantly lower odds of (re)bleeding during follow-up relative to no medical therapy (OR = 0.32, 95% CI: 0.14-0.73). A comparable reduction in hemorrhage risk was observed when statin monotherapy was evaluated against BB monotherapy (OR = 0.32, 95% CI: 0.11-0.95). The triple combination of statins, ATs, and BBs was associated with lower odds of follow-up hemorrhage compared with no treatment (OR = 0.24, 95% CI: 0.06-0.95).

For (re)bleeding during follow-up, the multivariate network meta-analysis indicated that triple therapy (statin + AT + BB) was ranked first in the SUCRA analysis, with a 48.3% probability of being the highest-ranked strategy, and was associated with statistically lower odds of (re)bleeding compared with no treatment (OR = 0.24, 95% CI: 0.06-0.95). Statin monotherapy ranked second in the SUCRA analysis (associated probabilities: 20.6% and 32.9%), with lower odds relative to both BB monotherapy (OR = 0.32, 95% CI: 0.11-0.95) and no treatment (OR = 0.32, 95% CI: 0.14-0.73). Conversely, BB monotherapy and no treatment consistently ranked lowest in the SUCRA analysis (probabilities of occupying the lowest-ranked position: 1.6% and 47.3%, respectively). Notably, BB monotherapy was associated with higher odds of hemorrhagic risk compared with AT monotherapy (OR = 2.41, 95% CI: 1.13-5.10) (Figures 4, 5). These SUCRA rankings represent probabilistic treatment hierarchies rather than definitive evidence of clinical superiority and should be interpreted cautiously given the predominantly observational evidence base.

**Figure 4:**
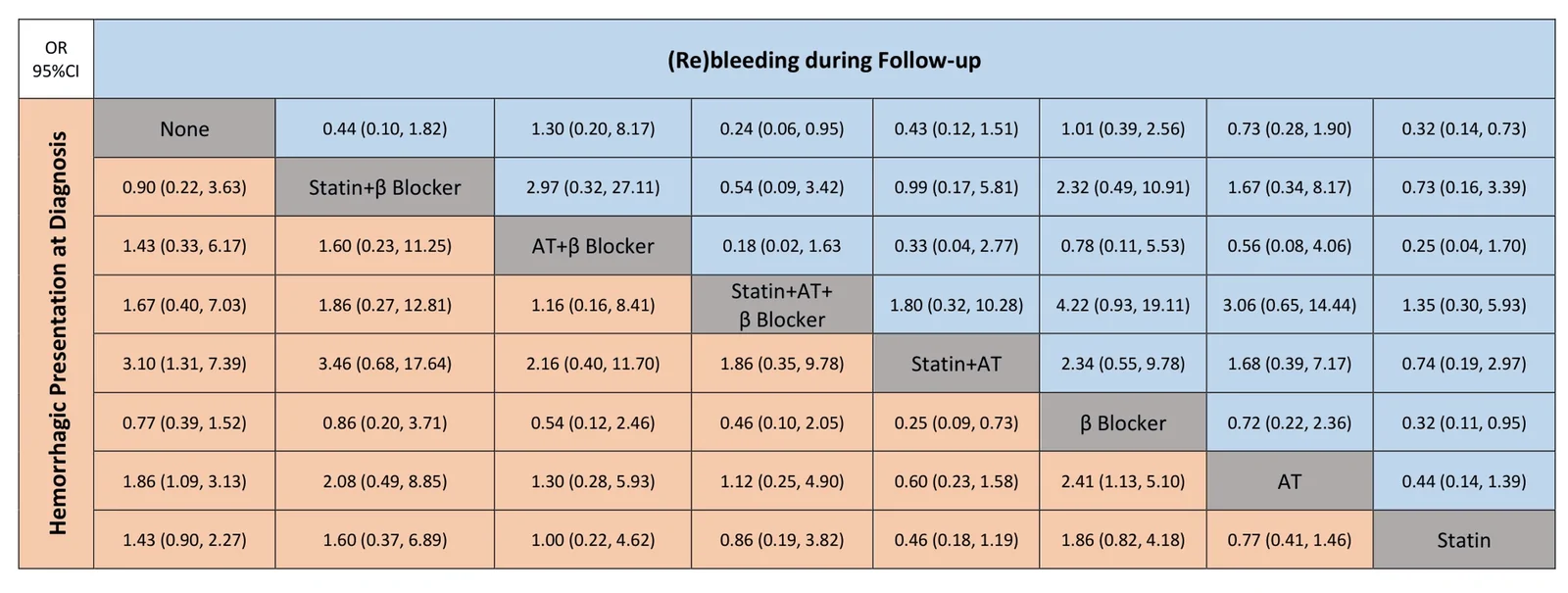
League table summarizing the relative treatment associations with hemorrhagic outcomes This matrix presents the comparative estimates for all direct and indirect (network) comparisons among the evaluated pharmacological regimens. The specific treatment regimens are listed along the central diagonal (gray cells) in a stepwise arrangement, ordered according to their overall ranking within the network hierarchy. The table is divided into two sections representing separate clinical endpoints: the upper-right area (blue cells) displays comparisons for (re)bleeding during follow-up, while the lower-left area (orange cells) displays comparisons for hemorrhagic presentation at diagnosis. Each cell at the intersection of a row and a column contains the pooled odds ratio (OR) with its 95% confidence interval (CI). The values represent the relative odds for the column-defining treatment versus the row-defining treatment. An OR below 1 indicates lower estimated odds of the corresponding hemorrhagic outcome for the treatment listed in the column, whereas an OR above 1 indicates higher estimated odds. Estimates are shown in bold when their 95% CI does not include 1, indicating a statistically significant difference between the compared regimens. AT: antithrombotic The figure was created by the authors using STATA version 16 (StataCorp LP, College Station, TX, USA).

**Figure 5:**
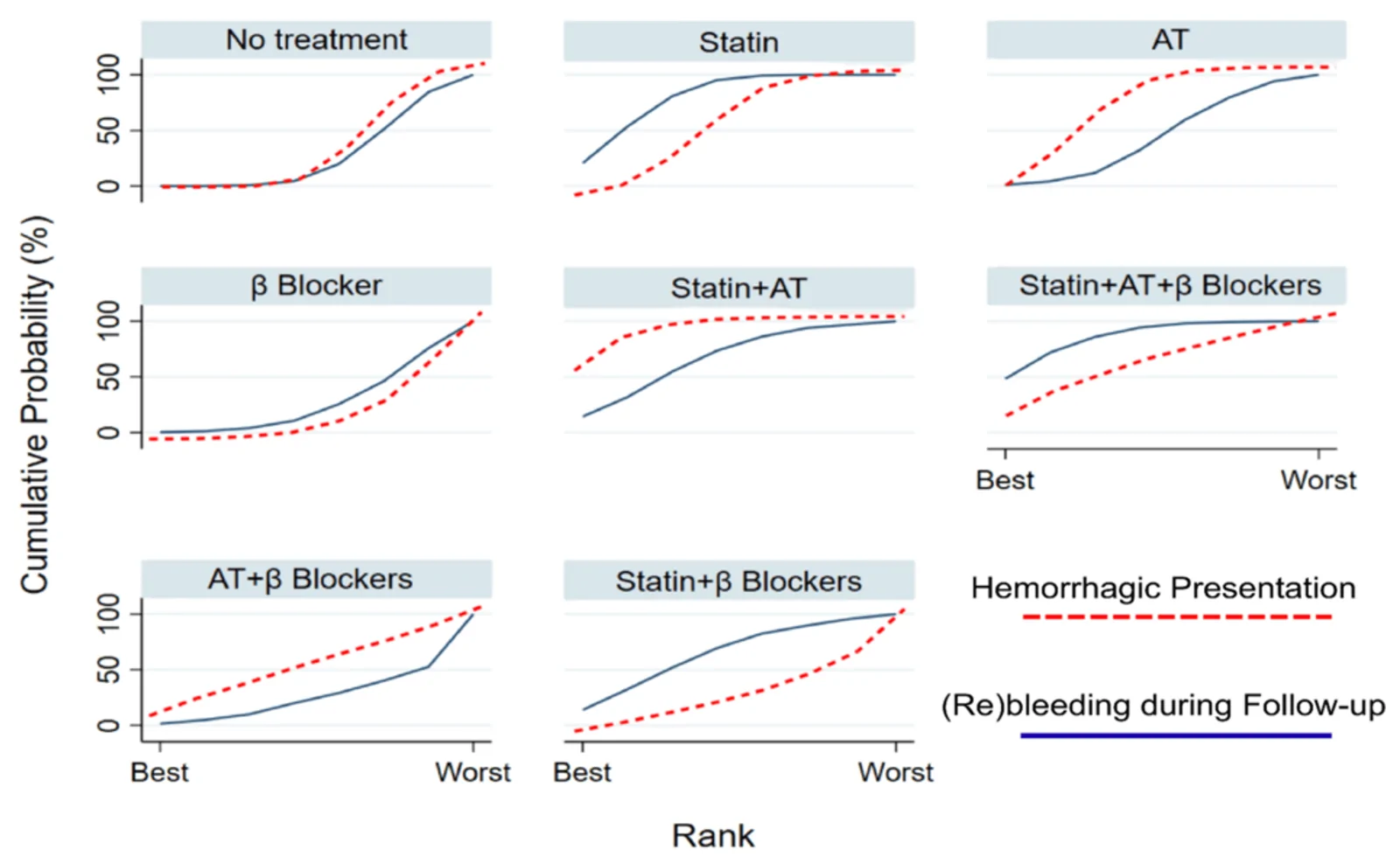
SUCRA illustrating the comparative ranking of pharmacological regimens for hemorrhagic outcomes This figure illustrates the cumulative ranking probabilities for each treatment strategy regarding two distinct clinical endpoints: hemorrhagic presentation (red dashed lines) and (re)bleeding during follow-up (solid blue lines). SUCRA plots summarize the treatment rankings by mapping the cumulative probability (Y-axis, from 0% to 100%) that a given intervention attains a specific rank or better (X-axis, ranging from "Best" to "Worst"). A curve shifted toward the top-left corner encompasses a larger area and indicates a greater probability of occupying a higher position in the treatment ranking, whereas a curve shifted toward the bottom-right corner indicates a greater probability of occupying a lower position. For (re)bleeding during follow-up, triple therapy (statin + AT + beta-blocker) was the highest-ranked regimen, with a 48.3% probability of being the highest-ranked strategy. Statin monotherapy also occupied a relatively high position in the cumulative ranking. In contrast, beta-blocker monotherapy and no treatment occupied lower positions in the ranking for bleeding outcomes. These SUCRA represent probabilistic treatment rankings and should not be interpreted as definitive evidence of clinical superiority, particularly given the predominantly observational evidence base. AT: antithrombotic; SUCRA: surface under the cumulative ranking curve The figure was created by the authors using STATA version 16 (StataCorp LP, College Station, TX, USA).

Secondary outcome: Results from seven included studies demonstrated that BB monotherapy was associated with higher odds of hemorrhagic presentation relative to AT monotherapy (OR = 2.41, 95% CI: 1.13-5.10). Compared with BB monotherapy, statin + AT was associated with significantly lower odds of hemorrhagic presentation (OR = 0.25, 95% CI: 0.09-0.73). Patients who had not received any of the investigated medications prior to diagnosis had higher odds of hemorrhagic presentation compared with those exposed to statin + AT combination therapy (OR = 3.10, 95% CI: 1.31-7.39). A similar pattern was observed when no treatment was compared with AT monotherapy (OR = 1.86, 95% CI: 1.09-3.13).

Regarding the risk of hemorrhagic presentation at diagnosis, the statin + AT combination achieved the highest SUCRA rank (57%). This was followed by AT monotherapy and other statin-based combinations. Although the evidence base for clinical presentation was less extensive than for follow-up outcomes, AT- and/or statin-containing strategies were associated with lower odds of hemorrhagic presentation compared with no treatment, whereas BB monotherapy and no treatment occupied lower positions in the SUCRA ranking. As with the primary outcome, these rankings should be interpreted as probabilistic treatment hierarchies rather than definitive evidence of clinical superiority.

Specifically, BB monotherapy showed a positive point estimate of risk relative to AT monotherapy (OR = 2.41, 95% CI: 1.13-5.10), although CIs for other BB combinations (e.g., statin + BB) were wide, reflecting uncertainty in these less frequently prescribed regimens (Figures 4, 5).

Discussion

Numerous studies have explored associations between exposure to ATs, statins, and BBs and hemorrhagic outcomes in patients with CCMs. However, to our knowledge, the comparative effectiveness of these medication classes has not previously been systematically assessed using a network meta-analysis framework. In this study, triple therapy (statin + AT + BB) was associated with lower odds of incident (re)bleeding during follow-up compared with no pharmacologic therapy (OR = 0.24, 95% CI: 0.06-0.95).

Statins

Preclinical evidence suggests that statins may benefit CCMs by inhibiting endothelial RhoA, whose signaling is upregulated by CCM-related mutations, compromising vascular barrier integrity [32]. The RhoA/ROCK pathway drives cytoskeletal remodeling, endothelial contractility, and barrier breakdown, effects that statins may partially mitigate by inhibiting RhoA activation and ROCK signaling (Figure 6) [12]. This disruption allows blood and hemoglobin byproducts to extravasate into adjacent brain tissue, promoting neoangiogenesis and lesion progression [33]. Given the oxidative and cytotoxic effects of free iron, targeting ROCK signaling and limiting iron deposition may constitute a mechanistically rational therapeutic approach [12,34,35]. In murine CCM models, statins reduced lesion growth and hemorrhage at doses comparable to those of selective ROCK inhibitors [35,36], though these models primarily reflect early lesion development rather than recurrent bleeding, limiting clinical extrapolation [23].

**Figure 6:**
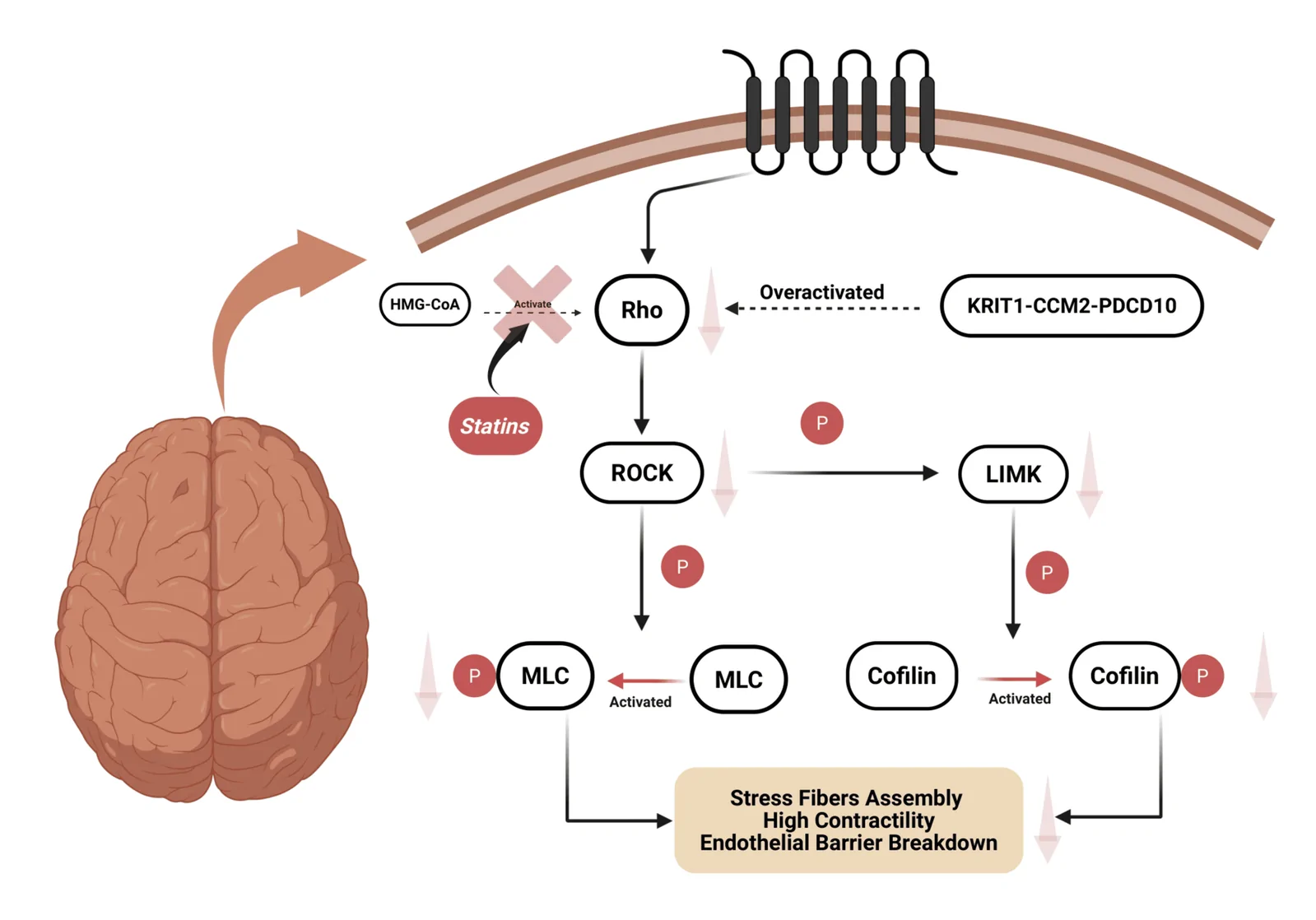
Mechanistic overview of statin-mediated modulation of RhoA/ROCK signaling in CCMs Statin-induced inhibition of HMG-CoA reductase, resulting in impaired RhoA prenylation and reduced ROCK activation. Downstream modulation of LIMK and cofilin phosphorylation attenuates stress fiber formation and endothelial barrier disruption associated with CCM lesions. CCM: cerebral cavernous malformation; HMG-CoA: hydroxymethylglutaryl-CoA; KRIT1 (CCM1): krev interaction trapped protein 1; LIMK: lim kinase; MLC: myosin light chain; PDCD10: programmed cell death; ROCK: rho-kinase The figure was created by the authors using BioRender.com.

Clinical findings are inconsistent, though observational studies have reported associations between statin exposure and lower annual hemorrhage rates or progressive neurological deficits [11,22]. Smaller cohorts likewise reported associations between statin exposure and lower hemorrhagic event rates with statin exposure [37,38]. Some studies indicate that combined statin and antiplatelet therapy may reduce unstable or hemorrhagic presentations, while statin monotherapy showed no independent effect [19,20]. Conversely, other series reported no significant link between statin use and hemorrhage risk [21,39]. Robust randomized data showed that atorvastatin did not significantly alter lesional iron burden or hemorrhage incidence over two years in symptomatic patients, indicating that current trial evidence does not support routine atorvastatin for preventing recurrent CCM hemorrhage [23]. However, a recent meta-analysis reported a reduction in the risk of follow-up hemorrhage with statin use, without a corresponding impact on diagnostic outcomes, further highlighting the heterogeneity of the available evidence [40].

Antithrombotics

The use of AT therapy in CCM patients remains debated [41-43]. Historically, concern has focused on the potential for AT agents to precipitate intracranial hemorrhage in structurally fragile CCM lesions; however, accumulating histopathological and epidemiological evidence challenges this paradigm. Ultrastructural studies show that CCMs consist of thin-walled, endothelium-lined vascular caverns that lack intervening brain parenchyma and often contain organized intraluminal thrombi. The characteristic low-flow hemodynamic environment may promote recurrent microthrombosis, impair venous outflow, increase local pressure, and trigger inflammatory and angiogenic cascades, thereby contributing to lesion instability and hemorrhage [44]. Against this mechanistic backdrop, inhibition of key platelet pathways, COX-1/TXA2 and ADP-P2Y12, may reduce thrombus-driven venous congestion and subsequent inflammatory destabilization in CCMs (Figure 7). In this context, Zuurbier et al. showed that long-term AT therapy was associated with a markedly reduced risk of intracranial hemorrhage or FND (adjusted hazard ratio (HR) 0.12), supported by pooled meta-analytic data [45]. Considered alongside histopathological evidence of intralesional thrombosis, these findings provide a potential mechanistic explanation for the observed association between AT exposure and lower hemorrhagic risk, although this mechanism has not been established clinically. Moreover, in our analysis, AT monotherapy was associated with significantly lower odds of hemorrhagic presentation compared with no baseline therapy.

**Figure 7:**
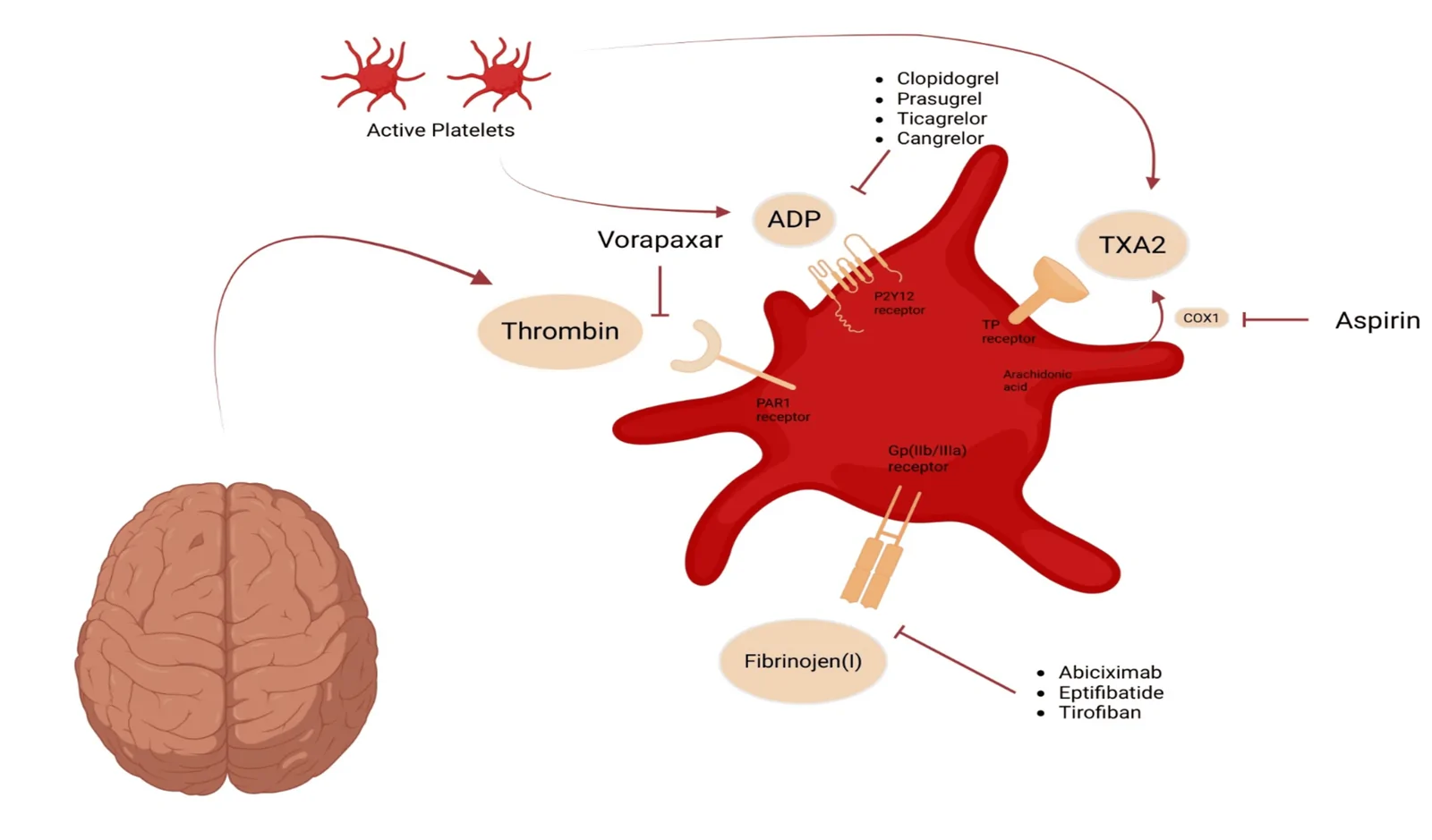
Mechanistic overview of platelet activation pathways and their pharmacological modulation in CCMs Central to hemostasis and thrombosis, platelet (thrombocyte) activation is mediated through several distinct receptor pathways, as depicted in the central cell diagram. Thrombin serves as a potent agonist by activating the protease-activated receptor-1 (PAR-1), a process selectively blocked by the antagonist vorapaxar. Concurrently, adenosine diphosphate (ADP) stimulates the P2Y12 receptor to promote platelet recruitment and aggregation; this critical pathway is inhibited by agents such as clopidogrel, prasugrel, ticagrelor, and cangrelor. Within the platelet, arachidonic acid is converted by cyclooxygenase-1 (COX-1) into thromboxane A2 (TXA2), which subsequently binds to the TP receptor to amplify activation-a cascade irreversibly inhibited by aspirin. Ultimately, these diverse intracellular signaling routes converge on the glycoprotein (GP) IIb/IIIa receptor, facilitating the cross-linking of platelets via fibrinogen binding. This final common pathway of aggregation is antagonized by abciximab, eptifibatide, and tirofiban. Through inhibition of platelet activation and aggregation, these agents may reduce thrombus formation and platelet-driven inflammatory signaling. In CCMs, which are prone to bleeding due to their abnormal vascular architecture, this may contribute to reduced lesion instability and hemorrhagic events. CCM: cerebral cavernous malformation The figure was created by the authors using BioRender.com.

Beta-Blockers

CCM has been redefined as a dynamic endothelial disorder marked by impaired barrier integrity and dysregulated angiogenic and inflammatory signaling, providing a broader mechanistic basis for pharmacologic intervention [33]. In this context, propranolol is mechanistically plausible, as beta-adrenergic blockade inhibits endothelial proliferation and migration and reduces angiogenic activity, suggesting a vascular-stabilizing effect [46]. Beyond antiangiogenic effects, beta-adrenergic blockade may reduce pulsatile hemodynamic stress and mechanosensory activation and intersect with the dysregulated RhoA-ROCK pathway implicated in CCM barrier failure, providing an additional rationale for lesion stabilization (Figure 8). Within this mechanistic context, interest in BBs for CCM was partly informed by the established efficacy of propranolol in infantile hemangiomas [47,48], which share certain vascular and histopathological features with CCMs [49,50]. However, evidence from infantile hemangiomas is indirect and should be regarded only as mechanistic rationale rather than clinical evidence of efficacy in CCMs. Despite the theoretical rationale, clinical evidence in CCMs remains limited. In our analysis, BB monotherapy was not associated with lower odds of hemorrhage at presentation or during follow-up, consistent with Goldberg et al. [51], who also found no association at either time point. Case reports and small series have described clinical improvement with propranolol [38,52,53], but these uncontrolled observations cannot establish treatment efficacy.

**Figure 8:**
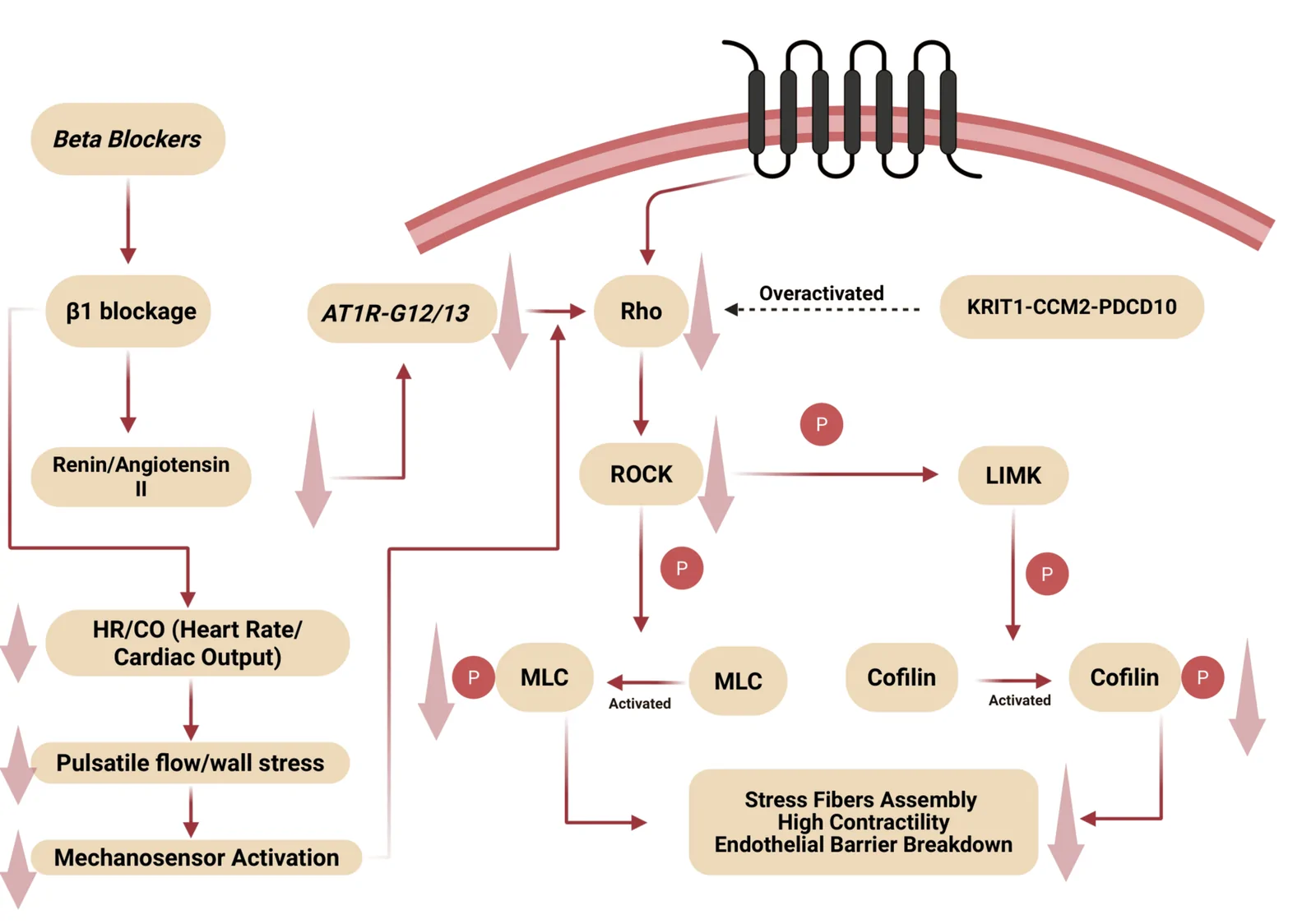
Mechanistic overview of beta-blocker-mediated modulation of RAAS and RhoA/ROCK signaling in CCMs BB-mediated suppression of RAAS activity and mechanosensory signaling, leading to reduced activation of Rho. Downstream attenuation of ROCK signaling results in decreased MLC phosphorylation, reduced actomyosin contractility, and stabilization of the endothelial barrier. AT1R: angiotensin II type-1 receptor; BB: beta-blocker; CCM2: cerebral cavernous malformation 2; CO: cardiac output; G12/13: G protein families 12 and 13; HMG-CoA: hydroxymethylglutaryl-CoA; HR: heart rate; KRIT1 (CCM1): krev interaction trapped protein 1; LIMK: lim kinase; MLC: myosin light chain; RAAS: renin-angiotensin-aldosterone system; ROCK: rho-associated protein kinase; PDCD10: programmed cell death 10 The figure was created by the authors using BioRender.com.

Other Medications

Female hormone therapy has also been investigated in relation to hemorrhage in CCMs, driven by its established associations with ischemic stroke and venous thromboembolism [54,55], and the hypothesis that intralesional thrombosis in CCMs may precipitate bleeding [56,57]. Available literature includes a case report suggesting a potential increase in hemorrhage risk with hormone therapy [58]. Several observational studies report an increased risk of both hemorrhagic presentation and subsequent hemorrhage in women receiving hormone therapy [59,60]. In contrast, Wildi et al. found no significant association with first or recurrent hemorrhage/FND events [61]. Vitamin D has emerged as a potential modulator of CM biology, particularly vascular integrity, with experimental (in vivo and in vitro) evidence suggesting that deficiency may promote endothelial dysfunction and increased vascular permeability [62]. Clinical evidence remains limited, though an early study reported lower serum 25-hydroxyvitamin D levels in patients with chronically aggressive disease. CM phenotype, characterized by early symptom onset, recurrent hemorrhages, or increased lesion burden, was not independently associated with hemorrhage alone [63]. Building on these observations, Flemming et al. found that patients with cerebral and spinal CMs presenting with hemorrhage had lower serum 25-hydroxyvitamin D levels than those without hemorrhagic presentation [64]. A recent systematic review has further synthesized these findings, suggesting that lower vitamin D levels may be associated with hemorrhagic presentations and increased disease severity, while vitamin D supplementation may be associated with a reduced risk of prospective hemorrhage; however, the evidence remains limited and predominantly observational [65]. These findings underscore the potential role of vitamin D as a modifiable factor in the pathophysiology of CCM, highlighting the need for more comprehensive prospective studies and RCTs.

Clinical Implications

Our findings are compatible with the following mechanistic hypothesis: ATs may mitigate intralesional microthrombus formation, statins may provide pleiotropic endothelial-stabilizing and anti-inflammatory effects, and BBs may reduce hemodynamic stress, which together could favor lesion stabilization. However, the apparent advantages of combination strategies in the network meta-analysis should be regarded as hypothesis-generating rather than evidence of pharmacological synergy, as the present analysis was not designed to estimate interactions between individual treatment components and the combinations were not evaluated in adequately powered randomized comparative trials. Observational studies have generally not demonstrated a consistent increase in CCM-related hemorrhage among patients exposed to these medications, and some have reported associations with a reduced risk of hemorrhage. Nevertheless, the findings are hard to interpret because of confounding by indication, differences in baseline hemorrhagic risk, heterogeneous treatment exposure and follow-up, and potential limitations of transitivity. Accordingly, the present findings should not be used to recommend statins, ATs, BBs, or their combinations specifically for the prevention of CCM-related hemorrhage. Prospective comparative studies and adequately powered randomized trials with standardized exposure definitions, hemorrhage outcomes, and follow-up are required to determine whether any of these observed associations represent true treatment effects.

Limitations

Although our meta-analysis has several strengths, including the inclusion of RCTs and prospective studies, a large pooled sample size with substantial person-years of follow-up, and a comprehensive systematic search strategy, it also has several limitations. Potential publication bias cannot be excluded, as studies with statistically significant results are more likely to be published than those with non-significant findings. Small-study effects may also be present, as studies with relatively small sample sizes tend to overestimate treatment effects. A significant proportion of the included studies were retrospective, which may have increased the risk of selection bias (e.g., patients at higher risk of hemorrhage may have been more likely to receive indicated medical therapies) and residual confounding (e.g., differences in comorbidities or baseline characteristics not uniformly adjusted across studies). Most treatments were prescribed for underlying cardiovascular or medical conditions rather than CCM-related indications, leading to substantial confounding by indication and potentially limiting the validity of indirect comparisons. Although adjusted estimates were preferentially used, the adjustment variables varied across studies, and unadjusted estimates were included when adjusted estimates were unavailable. Therefore, residual confounding cannot be excluded, particularly in the observational studies. Furthermore, the assessment of transitivity was limited by incomplete reporting of potential effect modifiers and the lack of treatment group-specific information for several relevant characteristics. These differences in clinical and methodological characteristics across studies may have affected the interpretation of indirect comparisons.

Reporting bias also cannot be excluded, particularly in cases involving subsequent surgical intervention, incomplete follow-up, or inconsistent outcome reporting, all of which may have affected the transparency of the reported results. Another limitation is incomplete reporting in some included studies. In a few cohorts, key descriptive statistics (e.g., mean age without a corresponding standard deviation (SD)) were not provided, limiting the precision of pooled mean ± SD estimates. In addition, some studies used broad or ambiguous treatment categories (e.g., grouping therapies outside the primary exposures of interest under “no treatment”) and did not report demographic characteristics stratified by pharmacologic therapy. As a result, potential exposure misclassification cannot be fully excluded, and it remains unclear whether additional medications were included within comparator groups.

## Conclusions

In this network meta-analysis incorporating mostly non-randomized studies, statin- and AT-based regimens were associated with lower hemorrhagic event rates in patients with CCMs, with triple-therapy combinations including BB ranking most favorably. However, these findings are substantially limited by confounding by indication, as treatments were prescribed for various underlying comorbidities rather than CCM indication, alongside potential transitivity violations across treatment nodes, residual unmeasured confounding, and imprecision from small sample sizes. Notably, the randomized AT CASH EPOC trial of atorvastatin did not demonstrate benefit on CCM-related bleeding, underscoring a discordance between observational associations and experimental evidence. Accordingly, the comparative treatment hierarchy should be considered exploratory and hypothesis-generating; these findings do not establish causality and should not currently alter clinical management of CCMs. Nevertheless, the observed associations may help prioritize pharmacological strategies for evaluation in future prospective studies. Adequately powered RCTs with standardized definitions of CCM-related hemorrhage, clearly defined treatment exposures, sufficient longitudinal follow-up, and stratification or adjustment for key baseline hemorrhagic risk factors are needed to determine whether these associations translate into clinically meaningful reductions in hemorrhage risk.

## Figures and Tables

**Figure 9:**
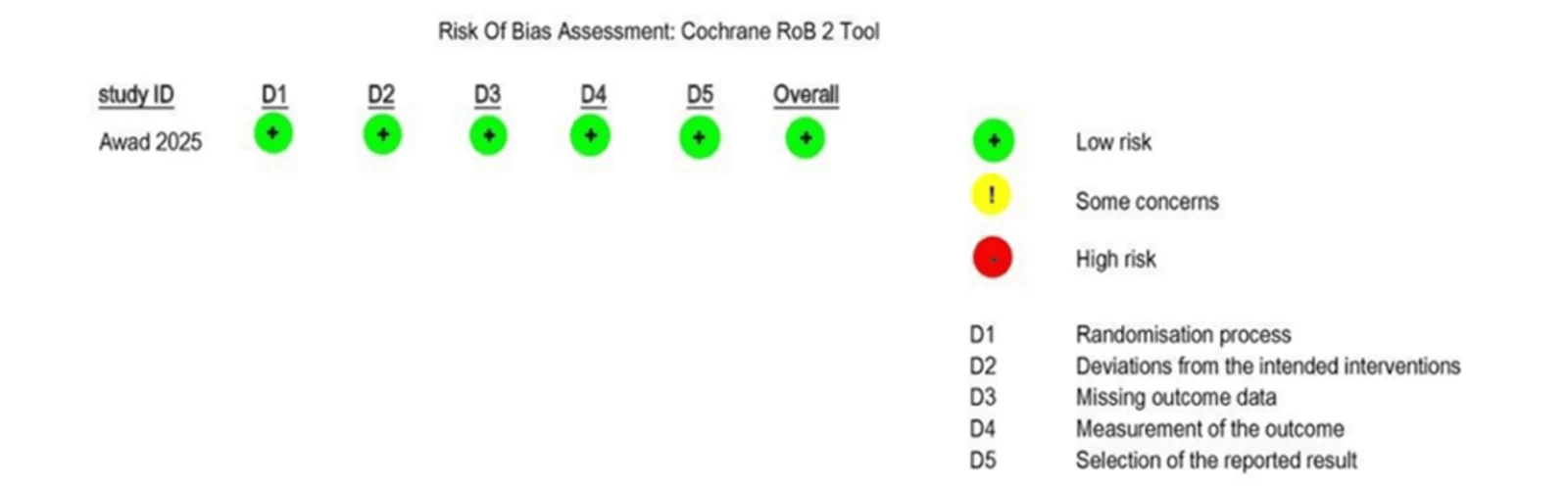
Risk-of-bias assessment for Awad et al. using the Cochrane RoB2 tool Domains assessed using the Cochrane Risk of Bias 2 (RoB2) tool [29]: (D1) Randomization process, (D2) Deviations from intended interventions, (D3) Missing outcome data, (D4) Measurement of the outcome, (D5) Selection of the reported result. Green (+) indicates low risk, yellow (!) indicates some concerns, and red (-) indicates high risk. The assessment pertains to Awad et al. [23].

## Appendices

Appendix A 

**Table 3:** Detailed search strategy used for all databases

Search strategy	
#1	((((((bleeding) OR (rebleeding)) OR (hemorrhage)) OR (hemorrhages)) OR (haemorrhage)) OR (haemorrhages)) AND (((((((((((((((((((((((((((((((((((((((((((((((((Cavernous Hemangioma, Central Nervous System) OR (Central Nervous System Cavernous Hemangioma)) OR (Cerebral Cavernous Hemangioma)) OR (Cavernous Hemangioma, Cerebral)) OR (Cavernous Hemangiomas, Cerebral)) OR (Cerebral Cavernous Hemangiomas)) OR (Hemangioma, Cerebral Cavernous)) OR (Hemangiomas, Cerebral Cavernous)) OR (Cavernous Hemangioma, Intracerebral)) OR (Cavernous Hemangiomas, Intracerebral)) OR (Hemangioma, Intracerebral Cavernous)) OR (Hemangiomas, Intracerebral Cavernous)) OR (Intracerebral Cavernous Hemangiomas)) OR (Intracerebral Cavernous Hemangioma)) OR (Cavernous Angioma, Central Nervous System)) OR (Cavernous Hemangioma of Brain)) OR (Brain Cavernous Hemangioma)) OR (Brain Cavernous Hemangiomas)) OR (Cerebral Capillary Malformations)) OR (Capillary Malformation, Cerebral)) OR (Capillary Malformations, Cerebral)) OR (Cerebral Capillary Malformation)) OR (Malformation, Cerebral Capillary)) OR (Malformations, Cerebral Capillary)) OR (Cavernous Angiomatous Malformations)) OR (Angiomatous Malformation, Cavernous)) OR (Angiomatous Malformations, Cavernous)) OR (Cavernous Angiomatous Malformation)) OR (Malformation, Cavernous Angiomatous)) OR (Malformations, Cavernous Angiomatous)) OR (Cerebral Cavernous Malformations)) OR (Cavernous Angioma, Familial)) OR (Angioma, Familial Cavernous)) OR (Angiomas, Familial Cavernous)) OR (Cavernous Angiomas, Familial)) OR (Familial Cavernous Angioma)) OR (Familial Cavernous Angiomas)) OR (Cerebral Cavernous Malformation)) OR (Cavernous Malformation, Cerebral)) OR (Cavernous Malformations, Cerebral)) OR (Malformation, Cerebral Cavernous)) OR (Malformations, Cerebral Cavernous)) OR (Familial Cavernous Malformation)) OR (Cavernous Malformation, Familial)) OR (Cavernous Malformations, Familial)) OR (Familial Cavernous Malformations)) OR (Malformation, Familial Cavernous)) OR (Malformations, Familial Cavernous))
#2	(((((((((((((((((((((((((((((((((((((((((((((((((((((((((((((((((((((((((((((((((((((((((((((((((((((((((((((((((((((((((((((((((((((((((((((((((((((((((((((((((((((((medical treatment) OR (medical management)) OR (Antithrombotic)) OR (Antithrombotics)) OR (Fibrinolytic Agent)) OR (Agent, Fibrinolytic)) OR (Fibrinolytic Drug)) OR (Drug, Fibrinolytic)) OR (Thrombolytic Agent)) OR (Agent, Thrombolytic)) OR (Thrombolytic Drug)) OR (Drug, Thrombolytic)) OR (Fibrinolytic Drugs)) OR (Thrombolytic Agents)) OR (Thrombolytic Drugs)) OR (Antithrombotic Agents)) OR (Agents, Antithrombotic)) OR (Antithrombotic Agent)) OR (Agent, Antithrombotic)) OR (Antithrombic Drug)) OR (Drug, Antithrombic)) OR (Antithrombic Drugs)) OR (Drugs, Antithrombic)) OR (Zocor)) OR (MK-733)) OR (MK733)) OR (MK 733)) OR (Synvinolin)) OR (Simvastatin)) OR (Atorvastatin)) OR (Hydroxymethylglutaryl CoA Reductase Inhibitors)) OR (Inhibitors, Hydroxymethylglutaryl-CoA Reductase)) OR (Reductase Inhibitors, Hydroxymethylglutaryl-CoA)) OR (HMG-CoA Reductase Inhibitor)) OR (HMG CoA Reductase Inhibitor)) OR (Statin)) OR (Statins)) OR (Inhibitors, HMG-CoA Reductase)) OR (Inhibitors, HMG CoA Reductase)) OR (Reductase Inhibitors, HMG-CoA)) OR (HMG-CoA Reductase Inhibitors)) OR (HMG CoA Reductase Inhibitors)) OR (Inhibitors, Hydroxymethylglutaryl-Coenzyme A)) OR (Hydroxymethylglutaryl-Coenzyme A Inhibitors)) OR (Inhibitors, Hydroxymethylglutaryl Coenzyme A)) OR (Inhibitors, Hydroxymethylglutaryl-CoA)) OR (Hydroxymethylglutaryl-CoA Inhibitors)) OR (Inhibitors, Hydroxymethylglutaryl CoA)) OR (Hydroxymethylglutaryl-CoA Reductase Inhibitor)) OR (Hydroxymethylglutaryl CoA Reductase Inhibitor)) OR (Reductase Inhibitor, Hydroxymethylglutaryl-CoA)) OR (Statins, HMG-CoA)) OR (HMG-CoA Statins)) OR (Statins, HMG CoA)) OR ((3R,5R)-7-(2-(4-Fluorophenyl)-5-isopropyl-3-phenyl-4-(phenylcarbamoyl)-1H-pyrrol-1-yl)-3,5-dihydroxyheptanoic acid)) OR (Lipitor)) OR (Atorvastatin Calcium)) OR (Atorvastatin, Calcium Salt)) OR (Atorvastatin Calcium Anhydrous)) OR (Liptonorm)) OR (Atorvastatin Calcium Hydrate)) OR (CI 981)) OR (CI-981)) OR (CI981)) OR (Atorvastatin Calcium Trihydrate)) OR (2-(Acetyloxy)benzoic Acid)) OR (Acetylsalicylic Acid)) OR (Acid, Acetylsalicylic)) OR (Solprin)) OR (Polopirin)) OR (Solupsan)) OR (Easprin)) OR (Zorprin)) OR (Acetysal)) OR (Acylpyrin)) OR (Colfarit)) OR (Ecotrin)) OR (Endosprin)) OR (Magnecyl)) OR (Micristin)) OR (Polopiryna)) OR (Aloxiprimum)) OR (Dispril)) OR (Blood Platelet Aggregation Inhibitors)) OR (Blood Platelet Antiaggregants)) OR (Antiaggregants, Platelet)) OR (Platelet Antiaggregants)) OR (Blood Platelet Aggregation Inhibitor)) OR (Blood Platelet Antiaggregant)) OR (Antiaggregant, Blood Platelet)) OR (Platelet Antiaggregant, Blood)) OR (Platelet Aggregation Inhibitor)) OR (Aggregation Inhibitor, Platelet)) OR (Inhibitor, Platelet Aggregation)) OR (Platelet Antiaggregant)) OR (Antiaggregant, Platelet)) OR (Platelet Antagonists)) OR (Blood Platelet Antagonists)) OR (Platelet Antagonist)) OR (Antagonist, Platelet)) OR (Blood Platelet Antagonist)) OR (Antagonist, Blood Platelet)) OR (Platelet Antagonist, Blood)) OR (Platelet Inhibitors)) OR (Platelet Inhibitor)) OR (Inhibitor, Platelet)) OR (Antiplatelet Agents)) OR (Antiplatelet Drugs)) OR (Antiplatelet Agent)) OR (Agent, Antiplatelet)) OR (Antiplatelet Drug)) OR (Drug, Antiplatelet)) OR (Protease-Activated Receptor-1 Antagonists)) OR (Protease Activated Receptor 1 Antagonists)) OR (PAR-1 Antagonists)) OR (PAR 1 Antagonists)) OR (Adrenergic beta Antagonists)) OR (beta-Antagonists, Adrenergic)) OR (Adrenergic beta-Antagonist)) OR (Adrenergic beta Antagonist)) OR (beta-Antagonist, Adrenergic)) OR (Adrenergic beta-Receptor Blockader)) OR (Adrenergic beta Receptor Blockader)) OR (beta-Receptor Blockader, Adrenergic)) OR (Blockader, Adrenergic beta-Receptor)) OR (beta-Adrenergic Antagonist)) OR (Antagonist, beta-Adrenergic)) OR (beta Adrenergic Antagonist)) OR (beta-Adrenergic Antagonists)) OR (Antagonists, beta-Adrenergic)) OR (beta Adrenergic Antagonists)) OR (beta-Adrenoceptor Antagonists)) OR (Antagonists, beta-Adrenoceptor)) OR (beta Adrenoceptor Antagonists)) OR (Adrenergic beta-Receptor Blockaders)) OR (Adrenergic beta Receptor Blockaders)) OR (beta-Receptor Blockaders, Adrenergic)) OR (Blockaders, Adrenergic beta-Receptor)) OR (beta-Adrenergic Receptor Blockaders)) OR (beta Adrenergic Receptor Blockaders)) OR (Blockaders, beta-Adrenergic Receptor)) OR (Receptor Blockaders, beta-Adrenergic)) OR (beta-Adrenergic Blocking Agents)) OR (Agents, beta-Adrenergic Blocking)) OR (beta Adrenergic Blocking Agents)) OR (Blocking Agents, beta-Adrenergic)) OR (beta-Adrenergic Blockers)) OR (beta Adrenergic Blockers)) OR (Blockers, beta-Adrenergic)) OR (beta-Blockers, Adrenergic)) OR (Adrenergic beta-Blockers)) OR (beta Blockers, Adrenergic)) OR (beta-Adrenergic Blocking Agent)) OR (Agent, beta-Adrenergic Blocking)) OR (beta Adrenergic Blocking Agent)) OR (Blocking Agent, beta-Adrenergic)) OR (beta-Adrenergic Receptor Blockader)) OR (beta Adrenergic Receptor Blockader)) OR (Blockader, beta-Adrenergic Receptor)) OR (Receptor Blockader, beta-Adrenergic)) OR (beta-Adrenoceptor Antagonist)) OR (Antagonist, beta-Adrenoceptor)) OR (beta Adrenoceptor Antagonist)) OR (beta-Adrenergic Blocker)) OR (beta Adrenergic Blocker)) OR (Blocker, beta-Adrenergic)) OR (Beta blocker)))
#3	#1 AND #2

Appendix B 

**Table 4:** AANS/CNS evidence classes and levels of recommendation The evidence classification and levels of recommendation were based on the American Association of Neurological Surgeons/Congress of Neurological Surgeons (AANS/CNS) criteria [30].

Evidence classification	
Class I evidence	Evidence from well-designed randomized controlled clinical trials (RCTs) and meta-analysis
Class II evidence	Evidence from well-designed comparative clinical studies (non-randomized cohort studies, case-control studies)
Class III evidence	Evidence from case series, case reports, comparative clinical studies, historical controls, clearly flawed randomized controlled clinical trials
Levels of recommendation	
Level I recommendation	Generally accepted patient-management strategies with a high degree of clinical certainty (Class I evidence on the direct clinical question or many Class II evidence when RCTs are not feasible)
Level II recommendation	Recommendations for patient management with clinical certainty (Class II evidence or a strong consensus of Class III evidence)
Level III recommendation	Patient management strategies for which the clinical utility is uncertain (Class III evidence with no consensus, inconclusive, or conflicting results)

Appendix C 

**Table 5:** Risk-of-bias assessment of the included observational studies using the Newcastle-Ottawa Scale (NOS) Stars (*) indicate fulfillment of the quality criteria for each domain: (1) representativeness of the exposed cohort, (2) selection of external controls, (3) ascertainment of exposure, (4) outcome not present at the start of the study, (5) comparability of cohorts (main factor), (6) comparability of cohorts (additional factor), (7) assessment of outcomes, (8) sufficient follow-up time, and (9) adequacy of follow-up. ^†^Scerrati et al. [24] is a case-control study. Since the cohort and case-control versions of NOS [27] are highly correlated, the table was constructed based on the corresponding items in the case-control version.

Study	Representativeness of the exposed cohort	Selection of the non-exposed cohort	Ascertainment of exposure	Outcome of interest was not present at start of study	Comparability of cohorts (main factor)	Comparability of cohorts (additional factor)	Assessment of outcomes	Sufficient follow-up time	Adequacy of follow-up	Total
Zuurbier et al., 2022 [11]	*	*	*	*	*	*	*	*	*	9
Marques et al., 2023 [19]	*	*	*	*	*	*	*	*	*	9
Chen et al., 2022 [21]	*	*	*	*	*	*	*	*		8
Santos et al., 2022 [22]	*	*	*		*	*	*	*		7
Scerrati et al., 2023^†^ [24]	*			*	*	*		*		5

Appendix D 

**Table 6:** Risk-of-bias assessment of Gomez-Paz et al. [20] using the NIH Quality Assessment Tool for observational cohort and cross-sectional studies ^†^The short labels in the table correspond to the full NIH Quality Assessment Tool [28] questions. All items in the NIH tool are conceptually correlated; therefore, abbreviated forms were used in the table for readability. The assessment was performed for the study by Gomez-Paz et al. [20]. CD: cannot determine; NA: not applicable; NR: not reported; NIH: National Institutes of Health

Criterion (NIH tool item)^†^	Judgment (yes/no/CD/NA/NR)*
Research question clear	Yes
Population defined	Yes
Participation ≥ 50%	CD
Same source population	Yes
Sample size justified	No
Exposure before outcome	CD
Timeframe sufficient	No
Exposure levels tested	CD
Exposure valid/reliable	Yes
Repeated exposure measure	No
Outcome valid/reliable	Yes
Blinding of assessors	CD
Lost to follow-up ≤ 20%	NA
Confounders adjusted	Yes

Appendix E 
